# Ancestry gaps in cardiovascular GWAS: a multi-database review of African representation in genomic studies

**DOI:** 10.3389/fgene.2025.1647176

**Published:** 2026-01-14

**Authors:** Diego A. Pomales-Matos, Mac Lyerly, Alejandro Rivera-Madera, Oswaldo L. Echevarría-Bonilla, Miguel Álvarez-Cortés, Saul E. Henriquez-Quiñones, Giselle M. Reyes-Sosa, Rafael A. Villanueva-Nogueras, Edwin G. Peña-Martínez

**Affiliations:** 1 Department of Biology, University of Puerto Rico – Rio Piedras Campus, San Juan, PR, United States; 2 Departmenf of Genetics, Stanford University, Stanford, CA, United States; 3 Department of Translational Neuroscience, Wake Forest University School of Medicine, Winston-Salem, NC, United States; 4 Department of Microbiology, Committee on Microbiology, University of Chicago, Chicago, IL, United States; 5 Department of Medicine, University of Puerto Rico – Medical Sciences Campus, San Juan, PR, United States; 6 Department of Medicine, Central University of the Caribbean, Bayamón, PR, United States; 7 Department of Genetics, Washington University in St. Louis School of Medicine, St. Louis, MO, United States

**Keywords:** African ancestries, cardiovascular diseases, genetic testing, genome-wide association studies, polygenic risk scores, population genomics

## Abstract

Cardiovascular diseases (CVDs) are the leading cause of death worldwide, claiming millions of lives each year. Genome-wide association studies (GWASs) have identified thousands of CVD-associated variants and have created the foundation for risk assessment and prevention through genetic testing. However, despite all the progress in understanding cardiovascular genomics, our genetic research and findings are overwhelmingly skewed towards individuals of European ancestry. This fact has limited our understanding and effectiveness for the diagnosis and treatment of CVDs in underrepresented populations, such as individuals of African ancestry. This gap is especially consequential because African ancestry populations harbor the greatest global genetic diversity, with variant frequencies and haplotypes that are often poorly captured by current reference datasets. In this review, we highlight recent efforts to understand the effectiveness of current tools in accurately diagnosing and treating CVDs in individuals of African ancestry compared to other populations. Additionally, we also performed a multi-database analysis to explore the persistent diversity gap in cardiovascular genetics. In doing so, we aim to raise awareness about the ancestry gaps faced in disease genomic research, supported by recent findings and the current landscape of our genetic databases.

## Introduction

Cardiovascular diseases (CVDs) remain the leading cause of mortality worldwide and are projected to increase by 90% over the next 25 years, with an estimated 35.6 million CVD-related deaths anticipated by 2050 (Li et al., 2023; Amini et al., 2021; Gaidai et al., 2023). In response to this growing burden, prevention strategies have increasingly emphasized the integration of genetic information into risk assessment tools. However, prediction of CVD risk remains imprecise due to the remaining ancestry gaps in biobanks and genome-wide association studies (GWASs) (Fernández-Rhodes, 2023). Despite global emphasis on diversity, over 90% of GWAS as of 2023 still focus on individuals of European descent, leaving other ancestry groups, especially African populations, significantly underrepresented (Zhang et al., 2023; Buniello et al., 2019). This imbalance highlights a systemic Eurocentric bias in genomic research; resources like the United Kingdom Biobank are widely used simply because large-scale genomic datasets for African and other underrepresented populations have not been developed to the same extent (Fatumo et al., 2022). As a result, polygenic risk scores (PRSs), which are derived mostly from these Eurocentric datasets, perform poorly in African individuals, whose unique genetic architecture is insufficiently captured (Ndong Sima et al., 2024; Adam et al., 2022). Paradoxically, Africa harbors the greatest genetic diversity in the world, yet many disease-relevant variants remain undiscovered due to limited inclusion in genetic research (Oni-Orisan et al., 2021; Tishkoff and Verrelli, 2003). This lack of representation reduces the clinical accuracy of PRSs and similar tools for African populations, hindering effective CVD risk prediction and widening disparities in precision medicine. These gaps are especially concerning given that African populations bear the highest global burden of cardiovascular disease and non-communicable disease mortality (Li et al., 2023; O’Sullivan et al., 2022; Minja et al., 2022).

A growing body of literature has examined the consequences of ancestral underrepresentation in genomic research, particularly related to CVD and African ancestry populations. Several reviews have already documented how Eurocentric study designs perpetuate disparities in both variant discovery and clinical translation. Bentley et al. (2020), for instance, highlighted the shortcomings of excluding African genomes, emphasizing that failure to include the world’s most genetically diverse populations limits the discovery of causal variants and reduces the applicability of genomic medicine (Bentley et al., 2020). Clarke et al. (2021) expanded on this point by demonstrating how racial disparities propagate across the cardiovascular genomics pipeline from genome assembly to clinical interpretation, ultimately limiting the utility of tools such as PRSs in non-European groups (Clarke et al., 2021). Earlier systematic evaluations, such as Kaufman et al. (2015), highlighted that genomic research had contributed relatively little to understanding the drivers of racial disparities in CVD, largely because key studies lacked adequate African ancestry representation (Kaufman et al., 2015). More recent reviews indicate that despite growing awareness, this issue persists. Ojewunmi and Fatumo (2025) argued that African genomic data remain essential for achieving equity in precision medicine and for building reference panels capable of capturing Africa’s enormous genetic diversity (Ojewunmi and Fatumo, 2025). Similarly, Jurado Vélez et al. (2025) emphasized that equitable cardiovascular genomics requires not only diverse sampling but also African-led research infrastructures, ethical frameworks, and sustainable capacity building (Jurado Vélez et al., 2025). Beyond broad assessments, several focused analyses have addressed specific CVD traits. For example, Bose et al. (2023) highlighted how genetic studies have struggled to explain racial disparities in CVD due to unbalanced sampling and the absence of fine mapping in African ancestry cohorts (Bose et al., 2023). Singh et al. (2021) further explored this challenge in their review of blood pressure and hypertension GWASs in African ancestry populations. They show that only a small fraction of known loci identified in European cohorts replicated in African cohorts, highlighting substantial population-specific biology (Singh et al., 2021).

Collectively, prior research underscores a consistent conclusion: African ancestry populations remain critically underrepresented across cardiovascular genomics research. This review builds on prior work by integrating cross-population GWAS findings with a multi-database quantitative analysis of cardiovascular genetic diversity, while also examining recent data on PRS performance, regulatory variation, and epigenetic differences across ancestries. We highlight the unique genetic architecture of African populations, the scientific and clinical consequences of their exclusion, and the structural, ethical, and logistical barriers that hinder their participation in global genomics research. Finally, this review outlines current initiatives addressing these challenges and proposes future strategies to enhance inclusion, foster equitable research, and improve precision medicine outcomes for African populations.

## The current state of genetic research in CVD

**FIGURE 1:**
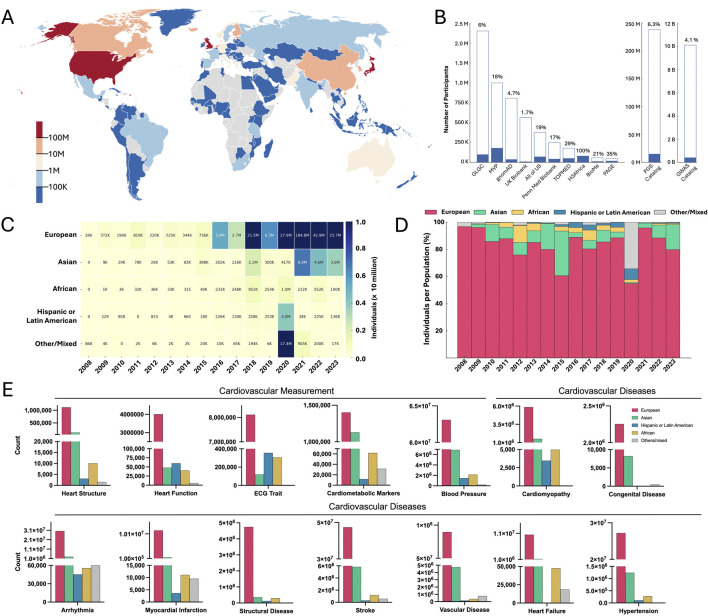
The state of cardiovascular genomic research. **(A)** Number of participants in GWASs from different regions worldwide. Note: Participants may be counted multiple times if their genome was used in more than one GWAS. The data used to generate the figure was downloaded from https://gwasdiversitymonitor.com. Legend is represented on a logarithmic scale. **(B)** Number of individual genomes sequenced per database. A fraction of African ancestry genomes is marked in blue, and percentages are at the top of each bar. **(C)** Number of participants from each ancestry in cardiovascular GWAS from 2008 to 2023. **(D)** Percentage of participants from each ancestry in cardiovascular GWAS from 2008 to 2023. Full information and details from **(A–D)** are available in Supplementary Material 1. **(E)** Total number of participants in GWASs for cardiovascular diseases and measurements to date. The color scheme for each population is the same as **(D)**. The source data was extracted from the GWAS diversity monitor database and is provided in the Supplementary Material 1. Note: The African ancestries displayed are a combination of sub-Saharan, African American, and Afro-Caribbean.

GWASs are a fundamental tool used globally to identify and annotate genetic variants associated with complex human traits and diseases (Buniello et al., 2019). The National Human Genome Research Institute–European Bioinformatics Institute (NHGRI-EBI) GWAS Catalog currently includes data from over 7,000 published studies, covering more than 5,000 diseases and traits. Increasing recognition of the value of GWASs has led to substantial growth in study sample sizes, resulting in more statistically significant associations across a wide range of phenotypes (Mills and Rahal, 2020; Abdellaoui et al., 2023). However, despite initiatives such as Human Heredity and Health in Africa (H3Africa) (Ramsay et al., 2016) and the African Genome Variation Project (Gurdasani et al., 2015) aimed at enhancing representation, GWASs remain predominantly Eurocentric (Figure 1A). As of mid-2025, individuals of European ancestry comprise 90.53% of all GWAS participants, while the representation of African ancestry populations has shown minimal progress over the past decade (Mills and Rahal, 2020). In over 15 years, the percentage of GWASs conducted primarily on individuals with European ancestry has only dropped 5% since the first quantitative review of ancestral diversity in GWAS by Need et al. in 2009 (Figure 1B) (Need and Goldstein, 2009; Corpas et al., 2025). This lack of diversity is not just limited to the broader landscape of genetic research, but is particularly pronounced in cardiovascular GWASs, where the underrepresentation of African and other non-European populations remains a persistent issue (Figures 1C,D; Supplementary Figure S1).

In 2021, Clarke et al. noted the lack of replication studies in coronary artery diseases (CAD), the largest studied cardiovascular phenotype, involving individuals of African and Latin American ancestry (Clarke et al., 2021; Singh et al., 2023; Matarin et al., 2008; Helgadottir et al., 2008; Cluett et al., 2009; Barad et al., 2024). Four years later, this observation largely remains accurate, as many genetic studies on CAD continue to rely exclusively on cohorts of European ancestry. For instance, Jain et al. (2024) investigated the role of sex hormones in CAD using only participants from the United Kingdom Biobank, all European descent. Similarly, Trenkwalder et al. (2025) explored the genetic overlap between CAD and aortic stenosis using data from sources like the Estonian Biobank and the FinnGen project, all limited to European ancestry (Trenkwalder et al., 2025). Although some researchers aim to include individuals of African ancestry, these efforts are hindered by small sample sizes, often limiting the statistical power and depth of analyses compared to those conducted in European populations (Sapkota et al., 2022; Rocheleau et al., 2024). Many studies investigating stroke, hypertrophic cardiomyopathy (HCM) and dilated cardiomyopathy (DCM) have also largely excluded African, Latin/Hispanic, or admixed populations (Traylor et al., 2017; Silva et al., 2023; Doumatey et al., 2023), reflecting the remaining ubiquitousness of underrepresentation across GWASs despite the growing burden of these diseases in African populations (Figure 1E) (Minja et al., 2022; Jordan et al., 2023; Keates et al., 2017).

## The impact of the diversity gap in cardiovascular genomics

The Eurocentric bias in GWASs has carried over into recent advances in cardiovascular genomics, particularly the development of polygenic risk scores (PRSs). PRSs estimate an individual’s genetic predisposition to disease by aggregating the weighted effects of multiple variants, offering clinical value for early prevention and intervention strategies (Bentley et al., 2020; Singh et al., 2023). However, because virtually all PRSs depend on GWASs to derive variant effect sizes (Xiang et al., 2024; Zhao et al., 2024), their performance is often compromised in non-European populations. This is due to the overwhelming predominance of European ancestry data in GWAS, which limits the applicability of PRSs in more genetically diverse groups, such as those of African ancestry (Fahed et al., 2021; Dikilitas et al., 2022). For example, a 2024 study by Topriceanu et al. found that polygenic scores for cardiovascular diseases performed significantly better (*p*-value < 0.025) in White European (AUC = 0.77, 95% CI [0.76, 0.78]) and South Asian (AUC = 0.74, 95% CI [0.70, 0.77]) individuals than in those of African ancestry (AUC = 0.66, 95% CI [0.61, 0.72]). Odds ratios (OR) were also greater for White Europeans (OR = 1.61, 95% CI [1.56, 1.66]) than for individuals of African ancestry (OR = 1.20, 95% CI [1.09, 1.32]) (Topriceanu et al., 2024). Similarly, Smith et al. (2024) reported that even when using summary statistics from the largest congenital heart disease (CHD) meta-analysis to date and the most ancestrally diverse cohort available, PRS performance for individuals of African ancestry still fell behind that of other groups (Smith et al., 2024). Hence, multi-ancestry PRSs developed using pruning and thresholding methods showed the strongest association with CHDs in individuals of European (OR = 1.65 95%, CI [1.59–1.72]) or South Asian ancestry (OR = 2.75, 95% CI [2.41–3.14]), while the lowest was identified in African ancestry (OR = 1.16, 95% CI [1.11–1.21]) (Smith et al., 2024).

Beyond differences in allele frequencies and effect sizes, a growing body of work across large biobanks has shown that PRS transferability is strongly constrained by cohort-specific LD structure and ancestry composition. Multiple biobank evaluations consistently demonstrate that PRS trained in predominantly European datasets lose substantial predictive accuracy when applied to African ancestry cohorts in resources such as United Kingdom Biobank, the Million Veteran Program, and other population-specific datasets (Martin et al., 2019; Duncan et al., 2019; Ding et al., 2023). By comparing ancestry representation in 733 polygenic scoring studies with global population estimates and evaluating cross-ancestry PRS performance, a study by Duncan et al. (2019) demonstrates both extreme European over-representation (∼460%) and substantial African and Latino under-representation, alongside reduced PRS accuracy in non-European groups (Duncan et al., 2019). Ding et al. (2023) similarly reported that PRS accuracy declines with increasing genetic distance in the ATLAS precision health biobank. Genetic distance was defined as the Euclidean distance between a target individual and the center of the training data in the principal component space. They found a strong negative correlation between genetic distance and PRS accuracy in African American individuals (R = −0.88). Moreover, those in the most genetically distant decile exhibited approximately four-fold lower PRS accuracy compared to individuals genetically closest to the training population, highlighting poor PRS transferability across ancestries (Ding et al., 2023). Importantly, reduced performance is not solely attributable to continental ancestry differences since even within the same ancestry label, PRS accuracy can vary considerably between biobanks due to differences in factors like sampling frameworks and environmental covariates (Mostafavi et al., 2020). Together, these observations reinforce that the diversity gap documented throughout this review is not merely a matter of unequal representation but also a mechanistic barrier that limits the portability of genetic risk models. They also highlight the need for either larger GWAS in underrepresented populations or methodological approaches that leverage multi-ancestry training and recalibration in target cohorts to ensure equitable risk prediction (Kurniansyah et al., 2023; Kachuri et al., 2024).

In parallel with limitations of PRS transferability, widely used clinical risk scores for CVD also demonstrate racial miscalibration, potentially aggravating inequities when combined with genomic predictors. Multiple evaluations of the Pooled Cohort Equations (PCE) and Framingham models document systematic over- or under-estimation of 10-year atherosclerotic CVD (ASCVD) risk in African ancestry individuals, with miscalibration errors exceeding ∼20–30% in some settings (Yadlowsky et al., 2018; DeFilippis et al., 2015; Khan et al., 2024). For example, Yadlowsky et al. found that PCE substantially misestimated 10-year ASCVD risk, with large errors for Black adults, a third of whom received extreme risk estimates that were less than 70% or more than 250% of those for White adults with matching risk profiles (Yadlowsky et al., 2018; DeFilippis et al., 2015). More recently, newer tools like the American Heart Association’s PREVENT equations and the UK’s QR4 algorithm have improved performance in their derivation cohorts. PREVENT, which is race-free, achieved strong discrimination and calibration in U.S. validation cohorts (Ade et al., 2024). QR4, optimized in very large United Kingdom primary-care records, also outperforms legacy United Kingdom risk scores (Hippisley-Cox et al., 2024). However, critical gaps remain: PREVENT’s external validation is based on U.S. data and aggregated racial categories that may not reflect the genetic and social diversity of continental African populations (Ade et al., 2024; Hippisley-Cox et al., 2024). Additionally, QR4 was developed and validated exclusively on United Kingdom data. While the authors highlight its superior performance compared to international risk scores, a lack of validation in non-UK populations suggests the need for external validation before broader international application (Hippisley-Cox et al., 2024). Because these clinical tools are increasingly used alongside PRS to guide preventive therapy, these limitations risk compounding bias in risk stratification, highlighting an urgent need for model recalibration in diverse cohorts and for ancestry-aware combination of clinical and genomic risk estimation (Khan et al., 2024).

The underrepresentation of African ancestry and other minoritized populations in genomic datasets not only hampers predictive accuracy but also reduces the clinical utility of these tools for identifying clinically significant variants, particularly those not shared across ancestries (Bentley et al., 2020; Jurado Vélez et al., 2025; Manrai et al., 2016). For instance, Landry and Rehm (2018) demonstrated that genetic testing for cardiomyopathy yielded a higher detection rate of pathogenic or likely pathogenic variants in White patients (29.0%; 1,314/4,539) compared to individuals from underrepresented minority (URM) groups—defined as African, Hispanic, Native American, and Pacific Islander ancestries—who had a detection rate of only 18.4% (155/842; χ^2^ = 39.8; *p*-value <0.001). Additionally, inconclusive results were significantly more frequent among URM patients (39.8%) than White patients (24.6%) (Landry and Rehm, 2018; Huang et al., 2024). More recently, Jordan et al. (2024) reported that patients of African ancestry had a markedly lower proportion of clinically actionable variants for dilated cardiomyopathy (8.2%, 95% CI, [5.2%–11.1%]) compared to patients of European ancestry (25.5%, 95% CI [21.3%–29.6%]), further underscoring the clinical consequences of ancestry-biased data representation.

Recognizing these limitations, the Polygenic Risk Methods in Diverse Populations (PRIMED) Consortium, an NIH-funded effort, was launched in 2024 to improve PRS performance across ancestries. PRIMED develops and benchmarks methods that account for cross-population differences in allele frequencies and LD structure, supported by a coordinated framework for harmonizing multi-ancestry datasets on cloud platforms such as AnVIL (Kullo et al., 2024). Recent work from the PRIMED Methods Working Group shows that multi-ancestry PRS models and ancestry-informed recalibration strategies improve prediction accuracy in underrepresented populations, including African ancestry cohorts (Kachuri et al., 2024). Through shared data resources and systematic evaluation across global populations, PRIMED remains one of the most active initiatives driving improvements in PRS transferability.

## Unique genetic architecture of African populations

**FIGURE 2:**
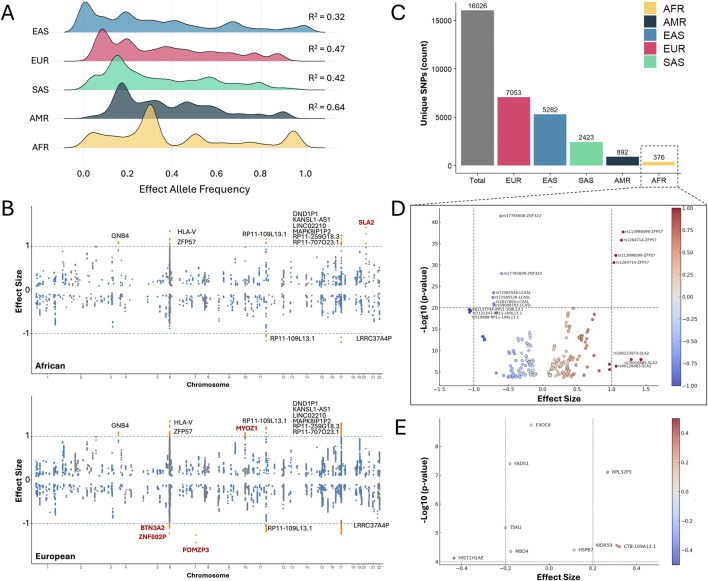
Ancestry-specific genetic architecture and regulatory mechanisms. **(A)** Effect allele frequency per population from expression quantitative trait loci (eQTL) in cardiac tissue (heart left ventricle and atrial appendage). The R^2^ value in each population is calculated based on the correlation with the African frequency. **(B)** Effect size of all SNPs in cardiac tissue in African (top) and European (bottom) ancestries. Genes in eQTL with an effect size >1.0 are colored orange and labeled. Genes unique for each ancestry LD block are labeled in red. **(C)** Number of unique cardiac GWAS single nucleotide polymorphisms (SNPs) per population. **(D)** Effect size of cardiac eQTL genes of African-specific SNPs. SNP-GENE combinations are labeled, with duplicate combinations representing different cardiac tissues. **(E)** Effect size of African-specific genes in cardiac eQTL. The data used to generate this figure was downloaded from Peña-Martínez et al. (2023) (Peña-Martínez et al., 2023; Peña-Martínez et al., 2025). A total of 16,026 CVD-risk variants that occur in at least one ancestry LD block were used to analyze differences in allelic frequencies and effect size across all five populations (see full list in Supplementary Material 3). Lead SNPs were identified from the GWAS catalog, followed by an LD expansion performed independently in each ancestry (R^2^ ≥ 0.80) using LDlinkR (Myers et al., 2020) and a cardiac eQTL analysis extracted from the GTEx database (Lonsdale et al., 2013). Output from LDlinkR includes the source population, allelic frequencies, and the effect size of each allele in different cardiac tissues.

Current precision medicine approaches for disease risk prediction and detection are significantly constrained by their inability to account for the extensive genetic heterogeneity observed in African ancestry populations. African genomes, on average, harbor approximately one million more genetic variants than non-African genomes (Sibomana, 2024; Zhang et al., 2022; Kamp et al., 2024). They also have lower linkage disequilibrium (LD), elevated allele frequency distributions for risk-associated variants, as well as the highest predicted 3D genome diversity (Kamp et al., 2024; Gilbertson et al., 2024). A study examining allele frequencies at 3,036 GWAS loci revealed that African populations have significantly higher frequencies of risk alleles compared to non-African populations (mean difference +1.15%, *p*-value = 0.0213, paired Wilcoxon signed-rank test) (Kim et al., 2018). We observed the same trend after performing a cardiac expression quantitative trait loci (eQTL) analysis of CVD-risk variants in African LD blocks (Figure 2A).

Differences between African and non-African populations extend beyond genetic sequences and variants, also encompassing regulatory elements and molecular interactions that govern gene expression and biological function. For example, *cis*-regulatory elements, such as enhancers, can exhibit population-specific activity. A study by Pettie et al. (2024) found that transcription factors (TFs) like NF-κB, JunD, and PU.1 showed signs of lineage-specific selection in enhancers but not in promoters (Pettie et al., 2024). This indicates that enhancers may play a key role in the differential regulation of gene expression across ancestries. The study underscores the utility of chromatin-level data in revealing hidden patterns of genetic variation shaped by evolutionary pressures. In many cases, such divergence is driven by variants that alter TF binding, pointing to population-specific regulatory mechanisms (Pettie et al., 2024; Tian et al., 2018; Husquin et al., 2018). Additional evidence comes from Patel et al. (2022), who analyzed data from African American and European American individuals in the Multi-Ethnic Study of Atherosclerosis (MESA). They reported significantly smaller gene expression effect sizes from trait-associated SNPs in African ancestry individuals compared to Europeans (slope = 0.85, 95% CI [0.81–0.89]) (Patel et al., 2022). This likely reflects differing patterns of gene-by-environment and gene-by-gene interactions, reinforcing the importance of incorporating diverse populations to better capture the full spectrum of regulatory variation in human genomics.

Epigenetic studies further reveal profound ancestry-specific differences that are often overlooked in Eurocentric research. Epigenetic influences shaped by environment and lifestyle, such as DNA methylation and non-coding RNAs, also appear to affect disease risk differently across ancestries. A review by Opare-Addo et al. (2024) found that these mechanisms may modulate stroke susceptibility in African populations, especially in response to psychosocial stressors (Opare-Addo et al., 2024). Despite this, only six studies have investigated the role of epigenetic regulation in stroke risk among individuals of African ancestry, revealing a stark gap in our understanding of ancestry-specific disease etiology. Additionally, broader evidence suggests that chromatin architecture and activity may differ by ancestry (Gilbertson et al., 2024), indicating that higher-order gene regulatory processes vary across populations in ways we are only beginning to understand.

Despite the rich genetic diversity originating from the African continent, GWASs have largely failed to capture this variation. We accessed cardiac tissue eQTL data on CVD GWAS variants and observed differences in effect size across ancestry LD blocks (Figure 2B; Supplementary Figure S2). Notably, while European ancestry populations lead in the discovery of unique single nucleotide polymorphisms (SNPs), African populations display a more than 40-fold lower count of registered cardiovascular GWAS SNPs, (Figures 2C,D). Conversely, cardiac eQTL analysis in African LD block also showed unique genes with genotype-dependent activity in cardiac tissue (Figure 2E) (Peña-Martínez et al., 2023; Peña-Martínez et al., 2025). The significant underrepresentation of African genomic variation helps explain the limited applicability of many existing PRSs in individuals of African descent, as trait-associated variants that are common in African populations may be rare or entirely absent in European-based GWAS cohorts.

Recent methodological work has focused on improving the transferability and fairness of PRS for underrepresented and admixed populations. Two papers by Martin and colleagues highlight the scope of this problem. Using 1000 Genomes data and GWAS summary statistics for multiple traits, Martin et al. (2017) demonstrated that PRS derived from single-ancestry GWAS are biased by demographic history and genetic drift, leading to poor performance across populations (Martin et al., 2017). Martin et al. (2019) further showed that current PRS are far more accurate in individuals of European ancestry, a consequence of Eurocentric GWAS sampling that may worsen health disparities if these scores are used clinically (Martin et al., 2019). Building on these observations, several methods have been recently developed to account for ancestry-related bias and heterogeneity. For example, PRS-CSx integrates GWAS summary statistics from multiple populations and integrates effect-size estimates through a Bayesian modeling framework, leveraging linkage disequilibrium (LD) diversity to improve prediction across ancestries (Ruan et al., 2022). Another method, GAUDI (Genetic Ancestry Utilization in polygenic risk scores for aDmixed Individuals), takes a different approach by using penalized regression to model ancestry-specific genetic effects while borrowing information across ancestral populations (Sun et al., 2024). Applied to data from the Women’s Health Initiative, GAUDI improved prediction for traits such as white blood cell count and C-reactive protein by over 60% in African-American samples, outperforming several existing methods, including PRS-CSx in some settings (Sun et al., 2024). FairPRS addresses the same challenge through invariant risk minimization (IRM). It uses an autoencoder-based framework that combines empirical and invariant risk objectives to generate ancestry-fair PRS or to debias existing scores. Evaluations on synthetic and United Kingdom Biobank data showed that FairPRS improves phenotype prediction while reducing ancestry-related bias (MacHado Reyes et al., 2023). Together, these studies illustrate complementary strategies that directly address the transferability and equity challenges described by Martin et al. They underscore the importance of incorporating ancestry-aware or multi-population models when deploying PRS in diverse clinical and research settings.

## Immigration, genomic research, and clinical outcomes

As of 2024, the number of international migrants has almost doubled since 1990, reflecting an increase from 2.9% to 3.7% of the world population (United Nations International Migrant Stock, 2024). Europe and North America are the regions receiving the most international migrants, and as the wealthiest areas of the world, they also have the most access to genetic testing and precision medicine. However, as previously discussed, the genetic databases upon which this healthcare depends do not equally benefit people of all ancestries.

Recent migration and diaspora movements have significantly shaped the distribution of African ancestry in global genomic cohorts, such that both historical forced migrations (e.g., the trans-Atlantic slave trade) and more recent voluntary mobility produce measurable admixture patterns and shifts in relatedness that directly affect association testing and ancestry-aware analyses in genomics. Understanding these patterns is necessary to contextualize the underrepresentation of African ancestry in genomic research. For example, analyses of African-American cohorts reveal that twentieth-century internal migrations within the United States (“Great Migration”) altered regional ancestry proportions and increased long-range genetic relatedness, showing how recent demographic mobility is reflected in genomic structures used for disease mapping and population studies (Baharian et al., 2016). Broader genomic investigations highlight that the underrepresentation of individuals with diverse ancestry in genomic research limits both discovery and translational impact, and that greater inclusion of such populations can advance understanding of genetic variation and health disparities (Fernández-Rhodes, 2023; Bentley et al., 2020). Moreover, considering African populations themselves is equally important, as their rich genetic diversity further informs global reference datasets. Within Africa itself, widespread genetic diversity shaped by ancient and recent movements and admixture underscores that both local and long-distance population flows contribute to the genetic landscape underpinning reference panels and risk prediction models (Busby et al., 2016). Finally, because sub-Saharan Africa has a comparatively young, working-age population and continued population growth relative to Europe and North America, demographic momentum will likely make African sources increasingly important for future international migration patterns, reinforcing the need for migration-informed genomic sampling strategies in global research (Mueller et al., 2019; Cottier, 2024).

Taken together, these demographic and genetic factors highlight why precision medicine must integrate population history to assess treatment effectiveness across ancestries accurately. Precision medicine aims to address variation in treatment effectiveness of diseases, and those of African descent can respond differently to CVD treatments compared to other ethnicities. Differences in effectiveness have been shown in the use of diuretics, calcium channel blockers, warfarin, and statins (including lovastatin and simvastatin) (Brewster et al., 2004; Dang et al., 2005; Shear et al., 1992; Krauss et al., 2008). Adverse reactions to angiotensin-converting enzyme (ACE) inhibitors have been demonstrably more prevalent in African Americans compared to white Americans, and the risk of hemorrhagic complications in patients with intracranial hemorrhage treated with thrombolytics shows a similar relationship (Wright et al., 2005; Mehta et al., 2014).

An unknown portion of this treatment discrepancy can be attributed to genetic variation. This challenge is exemplified in the pharmacogenomics of warfarin, where genes such as *CYP2C9* and *VKORC1*, often harboring unique mutations in African populations, significantly affect dosage requirements and risk of adverse drug reactions (Sibomana, 2024; Tata et al., 2020; Asiimwe et al., 2024). The *CYP2D6* gene, which affects the metabolism of hypertensive medications such as β-blockers, varies in frequency across the world (Lefebvre et al., 2007; Hamadeh et al., 2014). In fact, the allele frequency of *CYP2D6* does not only vary across global populations, but also within continental groups, with pronounced differences even among African lineages, including between native African populations and individuals of African descent in the American continent (Pettie et al., 2024). Similarly, for clopidogrel, a widely used anticoagulant, common variants in the *CYP2C19* gene among African individuals can reduce drug metabolism efficiency, thereby elevating the risk of cardiovascular ischemic events and mortality in comparison to non-African individuals (Tata et al., 2020; Asiimwe et al., 2024; Shuldiner et al., 2009). To further highlight this disparity, a study of heart failure (HF) in adults by Smith et al. (2010) found that the genome-wide significant SNP rs10519210, identified in individuals of European ancestry, was not associated with HF in those of African ancestry (Smith et al., 2010). Lastly, another study by the Consotium of Minority Population GWAS of Stroke (COMPASS), demonstrated how stroke disproportionately affects people of African ancestry, who face nearly twice the incidence and almost 3-fold higher mortality than European Americans. COMPASS addressed this gap by analyzing >22,000 African-ancestry individuals, identifying a genome-wide significant variant near HNF1A and validating most loci across independent cohorts, highlighting both shared and potentially novel stroke-risk regions (Keene et al., 2020). All of these differences, and others like it, are necessary to acknowledge for accurate identification of risk factors for various treatments. Due to the reliance of genetic medicine on large-scale databases to effectively and accurately identify risk-increasing variants, it is vital that more participants of non-European descent are included in genetic studies (Smith et al., 2016).

Clearly, statistics are worrisome, but the concern disproportionately weighs on the consciousness of those whose current efforts fail to properly serve. In the United States, for instance, African-Americans are more likely to experience uncertainty regarding personalized medicine and genetic testing outcomes compared to European-Americans (Singer et al., 2004; Suther and Kiros, 2009; Zimmerman et al., 2006; Bloss et al., 2010; Diaz et al., 2014). To truly fulfill the promise of precision medicine, clinical applications should be able to treat everyone, regardless of their ethnicity. On the contrary, failing to address the existing inequality of representation in genetic data will likely worsen already-present disparities. With the growing rates of immigration, the world is becoming increasingly transient, and populations are becoming less stratified. It is more important than ever to circulate cross-ancestry genomic data and improve clinical outcomes to maximize the potential of precision medicine. All in all, these findings stress the importance of diversifying genomic data sets to achieve a robust interpretation of genome-wide variation when generalizing findings and observations across ancestral populations.

## Diversification of GWAS with African ancestry data

**FIGURE 3:**
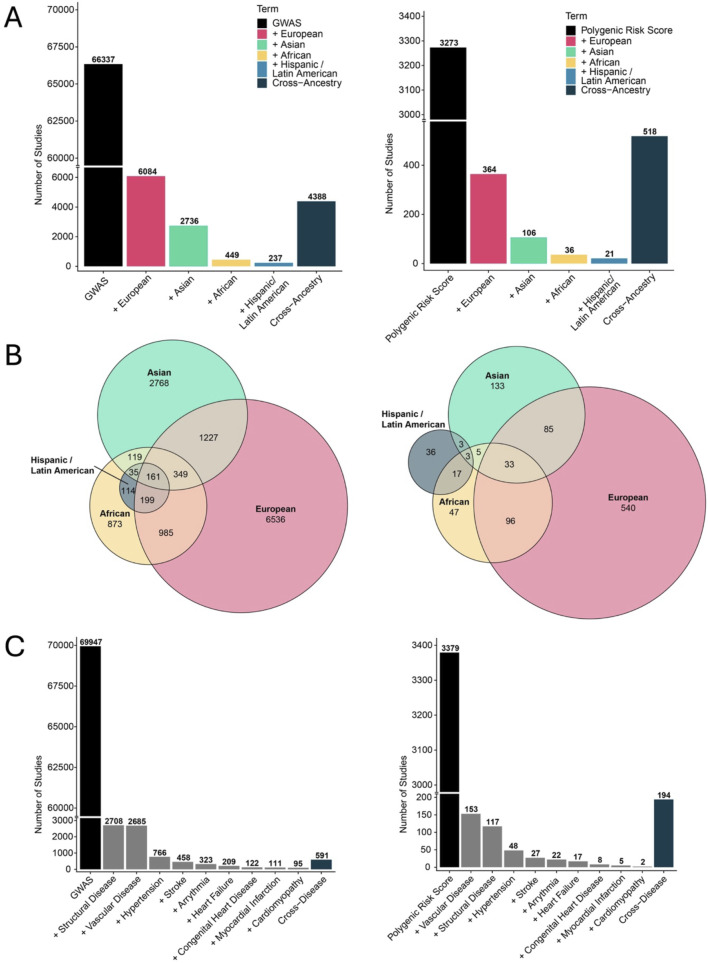
Racial disparities in GWAS and PRS research. **(A)** Number of GWAS (left) and PRS (right) publications registered in PubMed. The X-axis contains the terms used for the command-line-based query. The full list of publications and search terms is in Supplementary Material 4. **(B)** Venn diagram of PubMed IDs extracted from **(A)** showing GWAS (left) and PRS (right) publications of cross-ancestry analysis. **(C)** Number of GWAS (left) and PRS (right) publications on specific CVDs.

The imperative to increase diversity in genomic research extends beyond addressing health disparities in African populations as it also presents a strategic opportunity to enhance the resolution and generalizability of genomic studies worldwide. The current state of research in CVDs is heavily skewed towards European ancestries (Figures 3A,B). The number of publications on CVD GWAS on individuals of European ancestry is significantly higher than African (14-fold) and Hispanic (25-fold) ancestries. For PRS research, the magnitudes are 10-fold for African ancestry and 17-fold for Hispanic ancestry. Owing to their deep evolutionary history, African populations possess the most genetically diverse and ancient lineages among all human groups (Sibomana, 2024; Gomez et al., 2014). As such, greater inclusion of African ancestry individuals in genomic datasets is crucial for enriching the global catalog of human genetic variation. A striking example of this potential emerged in 2020 through the H3Africa initiative, which identified over 3.4 million genetic variants from whole-genome sequencing of just 426 individuals of African descent (Pereira et al., 2021; Choudhury et al., 2020). Despite this, African ancestry individuals represented only 2.4% of GWAS participants at the time yet contributed to 7% of disease-related genetic associations, underscoring their disproportionate value in variant discovery (Pereira et al., 2021). For some types of CVDs, there is a lack of genomic research, which compounds how our understanding is not only limited, but also heavily skewed towards European ancestries (Figure 3C). More recently, the inclusion of African ancestry populations has led to the identification of novel variants associated with a range of cardiovascular traits and diseases, including blood pressure regulation (Udosen et al., 2024), rheumatic heart disease (Machipisa et al., 2021), and cardiometabolic conditions (Choudhury et al., 2022), further highlighting the global scientific and clinical gains of diversifying genomics research.

The power of incorporating African ancestries is not just limited to increasing the diversity of datasets, but it’s also evident in the active improvement on the resolution and translational value of genetic studies for worldwide populations. As highlighted earlier, shorter LD blocks and greater haplotype diversity, characteristic of African genomes, offer a powerful advantage for fine-mapping disease-associated loci in GWASs (Kamp et al., 2024). This genomic architecture enables researchers to more precisely localize causal variants by minimizing the confounding effects of extended LD that often obscure association signals in European datasets. In a multi-ancestry GWAS of coronary artery calcification (CAC), the inclusion of African ancestry participants facilitated the discovery of eight novel loci and improved the resolution of known signals (Kavousi et al., 2023).

## Barriers to inclusion, ongoing initiatives, and solutions

The underrepresentation of individuals of African ancestry in genomics studies is not merely a statistical issue, but a reflection of deep-rooted challenges that hinder the development of genomic capacity across the African continent (Table 1). One of the most pressing barriers is inadequate funding in Africa, which represents the primary source for African ancestry data. Although sequencing costs have declined significantly since the Human Genome Project, many African researchers still cannot afford these services in regions where most graduate research is self-funded and economic hardship is widespread (Omotoso et al., 2022). As a result, the necessary research infrastructure, such as next-generation sequencing (NGS) and genotyping technologies, remains largely inaccessible across much of Africa (Bentley et al., 2020; Omotoso et al., 2022; Onywera et al., 2024; Olono et al., 2024). This financial limitation also contributes to a shortage of trained medical genetics professionals, further emphasizing the scarcity of local expertise (Onywera et al., 2024). Without adequate personnel, it becomes challenging to build a healthcare system that can support genomics-based research and clinical care (Kamp et al., 2021). Educational barriers add another layer to the problem since limited public awareness about the benefits of genomics contributes to low participation in research efforts and slows the integration of African ancestry data into broader genomic datasets (Kamp et al., 2021; Kamga et al., 2024). Ethical and cultural considerations present additional challenges. Concerns about informed consent, data ownership, and privacy must be addressed thoughtfully and transparently. Historical exploitation in medical research involving African populations has led to widespread mistrust and skepticism toward current initiatives, which continues to undermine public engagement (Kamga et al., 2024; Jackson et al., 2023; Amoakoh-Coleman et al., 2023).

**TABLE 1:** Summary of issues behind racial disparities in cardiovascular genomics and strategies to address them.

Issue	Short-term efforts	Long-term strategies	Key references
Eurocentric sampling and small AFR cohorts	Report ancestry composition and ancestry-specific results; include available AFR datasets in meta-analyses using multi-ancestry models	Fund and sustain large, well-phenotyped AFR and diaspora cohorts to balance global representation	Martin et al. (2019), Popejoy and Fullerton (2016)
Limited imputation & LD reference resources	Use multi-ancestry WGS panels (e.g., TOPMed); report per-ancestry imputation quality	Develop AFR-focused high-coverage reference panels and LD maps	Taliun et al. (2021)
Admixture and local ancestry complexity	Infer local ancestry (e.g., RFMix); adjust GWAS and PRS accordingly	Standardize local-ancestry-aware pipelines and benchmark across consortia	Maples et al. (2013)
PRS transferability and model bias	Evaluate PRS accuracy by ancestry; use ancestry-aware tools (e.g., PRS-CSx, GAUDI, FairPRS)	Build and validate PRS in large AFR datasets; promote development of transferable models	Ruan et al. (2022), Sun et al. (2024), MacHado Reyes et al. (2023)
Functional annotation and phenotype bias	Use diverse functional datasets when possible; harmonize phenotypes across cohorts	Expand AFR-specific functional genomics (eQTL, epigenomic) resources	GTEx Consortium (2020)
Capacity, governance, and equitable data sharing	Follow STREGA reporting; ensure equitable authorship and consent practices	Invest in African-led genomics capacity (e.g., H3Africa) and local data governance frameworks	Choudhury et al. (2020), Knoppers and Beauvais (2021)

Overcoming barriers to African representation in genomic research is essential for advancing global equity and scientific innovation. In recent years, several initiatives have emerged to promote inclusion, build research capacity, and embed ethical practices. The H3Africa initiative, launched in 2012, recognizes Africa’s vast genetic diversity as crucial to understanding human biology and addressing health disparities (Lumaka et al., 2022). It supports African-led projects, invests in sustainable infrastructure, and offers training in areas such as bioinformatics, grant writing, and ethical governance, while fostering locally informed and equitable research practices (Lumaka et al., 2022; Bentley et al., 2019). The 10-year H3Africa program, although coming to an end in 2022, supported three major biorepositories in Uganda, Nigeria, and South Africa, coordinating genomic and biospecimen collections across 30 African countries and maintaining a catalog that, as of June 2025, includes 19 studies, 37 datasets, over 23,000 biospecimens, and ∼120,000 participants (https://catalog.h3africa.org) (Abimiku et al., 2017). Its initiatives span a wide range of diseases, including cardiovascular, neurological, immune, reproductive, cancer, and infectious conditions, and have produced landmark resources such as the whole-genome sequencing of 426 individuals from 50 ethnolinguistic groups, which identified more than 3 million previously undescribed variants and 62 loci under strong selection (Choudhury et al., 2020). Building on this work, the planned 3 Million African Genomes (3MAG) project aims to generate a representative pan-African reference genome and a continent-wide clinical biobank, scaling efforts comparable to the United Kingdom Biobank’s 500,000-genome sequencing initiative (Wonkam, 2021). Similarly, the Harnessing Data Science for Health Discovery and Innovation in Africa (DS-I Africa) program, funded by the NIH Common Fund, also builds on the infrastructure and data-sharing foundations established by H3Africa to expand data science–driven health research across the African continent. It strengthens local capacity by supporting interdisciplinary research hubs, as well as open data platforms that enable African-led solutions to public health challenges (Adebamowo et al., 2023).

Similarly, the *All of Us Research Program*, led by the U.S. National Institutes of Health, enhances African ancestry representation by collecting diverse whole-genome sequences, thereby improving the accuracy of PRSs for underrepresented populations (Jurado Vélez et al., 2025; Gouveia et al., 2025; Tsuo et al., 2024). Likewise, the African Genomic Medicine Portal (AGMP) curates African-specific data to improve population sampling and geographic granularity in genomic medicine research. The Africa Pathogen Genomics Initiative (PGI), established by the Africa CDC in 2020, strengthens lab and bioinformatics capacity through an African-owned data platform (Othman et al., 2022). The Together for CHANGE (T4C) initiative, launched in 2023, promotes inclusive innovation through community engagement, public-private partnerships, and equitable healthcare development (Hildreth and Shanker, 2024). Finally, as briefly mentioned earlier, efforts like COMPASS further expand representation by coordinating stroke GWAS to provide ancestry-informed insights into cerebrovascular risk (Keene et al., 2020). Collectively, these efforts encourage investigators working with African genomic data to share their datasets through public repositories while also educating research participants about the importance of sharing aggregate frequency data and increasing funding to support large-scale generation of African genomic data.

## Conclusion

Equitable representation in cardiovascular genomics is not only a matter of scientific rigor but also of global health justice. African populations, while bearing a disproportionate burden of CVD and possessing unparalleled genetic diversity, remain vastly underrepresented in GWASs and related studies. This lack of inclusion limits the clinical validity of tools like PRSs and contributes to persistent disparities in disease prediction, prevention, and treatment. Enhancing diversity in genomics, particularly through the inclusion of African ancestry data, holds immense potential to improve the resolution and generalizability of genetic discoveries for all populations. Addressing the structural, economic, and ethical barriers to African participation is critical to achieving these goals. Initiatives like H3Africa, AGMP, and Together for CHANGE represent important strides toward building capacity, fostering ethical engagement, and promoting sustainable, African-led research. Moving forward, a concerted global effort is needed to support infrastructure development, facilitate data sharing, and educate communities on the value of genomics. In the US, many of these efforts and solutions are only possible through the continued support of federal funding agencies (NIH, NSF), as well as other global charitable foundations, such as Wellcome Trust. Only by closing the diversity gap can we realize the full promise of precision medicine and ensure that its benefits extend to all people, regardless of ancestry.
