# Supplementary material for: Ancestry gaps in cardiovascular GWAS: a multi-database review of African representation in genomic studies
Source: Front Genet. 2026 Jan 14;16:1647176. doi: 10.3389/fgene.2025.1647176 (PMC12844564; doi:10.3389/fgene.2025.1647176)
Supplement: Supplementary file 4 [file DataSheet1.docx]

**Supplementary Figures**

**Supplementary Figure 1**: Number of GWAS participants by year. General (top) and cardiovascular (bottom) GWAS participants are displayed cumulatively by year. Total numbers are displayed in the legend of each plot.

**Supplementary Figure 2**: Population-specific cardiac eQTL analysis. Each dot represents a unique SNP-gene pair in eQTL in cardiac tissue (heart left ventricle and atrial appendage). Cardiac eQTL analysis was performed separately in each population LD block. Genes not present in African blocks are highlighted in red. Ancestry-specific genes are highlighted in gree.
